# The Oxidative Stress in Knee Osteoarthritis Patients. An Attempt of Evaluation of Possible Compensatory Effects Occurring in the Disease Development

**DOI:** 10.3390/medicina55050150

**Published:** 2019-05-16

**Authors:** Marek Paździor, Małgorzata Kiełczykowska, Jacek Kurzepa, Dorota Luchowska-Kocot, Joanna Kocot, Irena Musik

**Affiliations:** 1 Traumatic-Orthopaedic and Spine Surgery Ward, Independent Public Health Care Centre in Puławy, 24-100 Puławy, Poland; marpazd@op.pl; 2 Chair and Department of Medical Chemistry, Medical University of Lublin, 20-093 Lublin, Lublin Province, Poland; jacek.kurzepa@umlub.pl (J.K.); dorota.luchowska-kocot@umlub.pl (D.L.-K.); joanna.kocot@umlub.pl (J.K); irena.musik@umlub.pl (I.M.)

**Keywords:** knee OA, total antioxidant status, superoxide dismutase, glutathione peroxidase, catalase, gender

## Abstract

*Background and Objective*: Osteoarthritis (OA) is a disorder of the musculoskeletal system resulting in worsening of life condition. The research revealed the involvement of oxidative stress into both OA pathogenesis and the effects of therapeutic agents applied in OA cases. The activities of the most important antioxidant enzymes, namely superoxide dismutase (SOD), glutathione peroxidase (GPx), catalase (CAT) and total antioxidant status (TAS), in blood of the knee OA patients were studied, with the aim of clarifying which enzymatic antioxidants are involved into osteoarthritis (OA)-related oxidative stress and whether any compensatory effects occur. The results were additionally analyzed with regard to gender. *Methods*: Whole blood SOD (U/mL), plasma GPx (U/L) and CAT (U/mL) activities as well as plasma TAS (mmol/L)) in knee OA patients were investigated. Sixty-seven patients (49 females and 18 males) with primary knee OA were enrolled. The control comprised 21 subjects (10 females and 11 males) free of osteoarthritis or inflammation. *Results*: TAS was decreased in OA subjects (4.39 ± 0.53 vs. 4.70 ± 0.60), with this effect being more significant in OA females (4.31 ± 0.51 vs. 5.02 ± 0.54). GPx was depressed in all OA patients (518 ± 176 vs. 675 ± 149). In both genders, GPx was decreased, significantly in males (482 ± 185 vs. 715 ± 105). SOD was decreased in all OA patients (109 ± 32 vs. 127 ± 42). CAT showed no difference in all OA subjects vs. control, while in OA females it was depleted (20.2 (11.6–31.6) vs. 38.5 (27.9–46.6)) and in OA men it increased (26.9 (23.3–46.5) vs. 14.0 (7.0–18.6)). *Conclusions*: The obtained results suggest that in men some compensatory mechanisms towards OA-related oxidative stress occurred. Based on the obtained data, the introduction of antioxidant supplements into OA therapy could be suggested with further research concerning the choice of agents.

## 1. Introduction

Osteoarthritis (OA) belongs to commonly occurring disorders of the musculoskeletal system. Its consequence is the constant worsening of condition, concomitant with pain as well as with joint stiffness and up to date no sufficient therapy resolving it has been found. The research on the processes involved into its pathogenesis, development and possible cures is still being continued [1,2,3,4,5,6]. The knee appears to be one of the most frequent sites affected by OA [7,8].

The OA can be of primary or secondary (caused by metabolic disorders or trauma) origin. The OA incidence is also connected with obesity, genetic factors and environmental factors, and has been found to be more frequent in women than in men. It has also been found to be intensifying with age [9,10,11,12,13,14].

Oxidative stress is a disorder of the balance between reactive oxygen species (ROS) and antioxidant defense [15]. ROS are the active molecules or radicals generated in the process of incomplete reduction of oxygen molecule or during further ROS interactions [16]. Although these play an important role in the human organism such as being involved in pathogen destruction or cellular signaling regulation [16,17], their excessive production may result in severe disturbances including deterioration of cellular membranes, proteins or DNA [16,18]. For these reasons, maintaining the antioxidant defense of the body, consisting of substances called antioxidants, capable of neutralizing ROS, is a crucial functional issue. Organisms develop a multitude of several antioxidants, of both macromolecular (enzymatic) nature (superoxide dismutase (SOD), seleno-dependent glutathione peroxidase (GPx) and catalase (CAT)) as well as low-molecular ones (e.g., reduced glutathione (GSH), vitamin C (ascorbic acid, AA), uric acid (UA), and tocopherol (vitamin E)) [2,12,16,17,19,20].

In human and animal studies, the involvement of oxidative stress into both OA pathogenesis [14,21,22,23] and the effects of therapeutic agents applied in OA cases [1,2,3,24] has already been confirmed. Moreover, the beneficial effects of substances acknowledged as containing natural antioxidants, such as pomegranate juice [4] or sesame powder [15], on oxidative and lipid profile parameters in OA patients has been reported. The association of pain alleviation resulting from the physical therapy with changes of oxidative parameters, observed in OA subjects, may be regarded as another proof of the relationship between oxidative stress and the OA course [11]. Furthermore, according to Olszewska-Słonina et al., in patients undergoing arthroplasty due to hip and knee OA of varied etiology, after 10-day-postoperative period, the alterations of chosen antioxidant enzymes were reported [25].

However, there is no consensus among researchers which elements of antioxidant barrier are the most susceptible to oxidative processes occurring during OA, as the results of published studies seem inconsistent, particularly with regard to enzymatic studies [21,22,25,26,27]. The clarifying of this question could contribute to the development of new therapeutic strategies involving the introduction of chosen antioxidants, e.g., selenium preparations, as adjuvants alleviating OA severity. Moreover, the reports on the analysis of the obtained data with regard to gender are scarce, although the differences in frequency of OA incidence between males and females have been emphasized [10,12]. Considering the presented facts, we studied the activity of the three most important antioxidant enzymes, SOD, GPx and CAT, as well as the total antioxidant status (TAS) in the blood of the knee OA patients. As the possibility of the existence of some compensatory mechanisms, e.g., an increase in any antioxidant in a response to depletion of others as an attempt towards alleviation of OA-induced damage of antioxidant defense, has already been suggested and investigated [20,21], we analyzed the obtained results with regard to this question. The obtained data were additionally analyzed considering the gender.

## 2. Materials and Methods

### 2.1. Patients

The current study included 67 patients of mean age 69.2 ± 8.3 years (49 females, mean age, 70.0 ± 7.9; and 18 males, mean age, 66.8 ± 9.2), admitted to the Trauma and Orthopaedic Ward of Independent Public Health Care Centre in Łęczna, Lublin Province, Poland, due to primary knee osteoarthritis and qualified for surgery. The knee osteoarthritis diagnosis was based on medical history; physical examination; and X-ray, ultrasound examination or computed tomography scan of knee joint.

The control group comprised 21 subjects with the mean age of 56.4 ± 19.1 years (10 females, mean age 71.0 ± 15.3; and 11 males, mean age 43.2 ± 10.8) free of osteoarthritis, other inflammatory processes or tumor.

All subjects participating in the study were provided with the proper information concerning the purpose of the study. Written consent was obtained from all subjects enrolled to the experiment prior their inclusion in the study. The study was approved by Bioethical Board of Medical University of Lublin (acceptance KE-0254/11/2016) and performed in accordance with the ethical standards laid down in the Declaration of Helsinki and its later amendments.

Inclusion criteria were as follows: limited physical activity, degeneration changes stated in the image, the overall condition allowing surgery under extrameningeal or general anesthesia.

Exclusion criteria were: body mass index (BMI) > 30.0 kg/m^2^; cancer; infection of joint or its region; disorders of blood clotting; limb ischemia as well as varicose veins with thrombophlebitis complications; smoking; and taking antioxidant supplements or pharmacological preparations containing vitamins A, C, and E or selenium.

The patients were given the following pharmacological agents: nonsteroidal anti-inflammatory drugs as ibuprofen or meloxicam, directly or in continued regime, depending on the pain intensity, as well as additional adjuvants (collagen, glucosamine, chondroitin, hyaluronic acid preparations, steroids, and magnesium sulfate).

The studied groups were denoted in a following way: Control, the control group; C-male, male control subjects; C-female, female control subjects; OA patients, patients with knee osteoarthritis; OA-male, male patients with knee osteoarthritis; and OA-female, female patients with knee osteoarthritis.

The samples of fasting blood were drawn during standard diagnostic examinations.

### 2.2. Biochemical Procedures

The samples of fasting venous blood (taken after 12-h overnight fasting) were drawn from control subjects and patients before surgery using test-tubes with an anticoagulant. Plasma samples were separated by centrifugation at 3000 rpm for 10 min and stored at −20 °C for further assays.

In whole blood, SOD activity was determined by a spectrophotometric method using diagnostic RANSOD (Randox Ltd., Crumlin, UK) kit and expressed in U/mL of whole blood.

Plasma TAS value was measured by a spectrophotometric method using diagnostic kit by Randox Ltd., Crumlin, UK and expressed in mmol/L.

Plasma GPx activity were measured by a spectrophotometric method using RANSEL (Randox Ltd., Crumlin, UK) diagnostic kit and expressed in U/l.

Plasma CAT activity was determined by a spectrophotometric method described by Aebi [28] according to Khosrowbeygi and Zarghami [29] and expressed in U/mL.

The assays were performed using SPECORD M40 (Carl Zeiss, Jena, Germany) spectrophotometer.

The following diagnostic parameters were determined using SYSMEX XT-4000i apparatus (Sysmex Polska, Warsaw, Poland): white blood cells (WBC); red blood cells (RBC); hemoglobin (HGB); hematocrit (HCT); mean corpuscular volume (MCV); mean corpuscular hemoglobin (MCH); mean corpuscular hemoglobin concentration (MCHC); red blood cell distribution width, coefficient of variation (RDW-CV); platelets (PLT); platelet distribution width (PDW); mean platelet volume (MPV); platelet large cell ratio P-(LCR); and plateletcrit (PCT).

### 2.3. Statistics

The statistical analyses were performed using STATISTICA 12 software. For verifying the normality of data distribution, Shapiro–Wilk test was used.

The differences between the OA patients and Control groups were estimated using the *t*-Student test (for normally distributed variables) or the U Mann–Whitney test (for non-normally distributed variables).

The differences between the OA-male, OA female, C-male and C-female groups were estimated using a one-way analysis of variance (ANOVA), followed by Tukey’s honest significant difference test HSD (for normally distributed variables) or the Kruskal–Wallis one-way analysis of variance test (for non-normally distributed variables).

As there was a considerable difference in age between male groups (OA-male and C-male), multi-way analysis of variance ANOVA/MANOVA for these groups was performed. The same analysis was performed for female groups (OA-female and C-female). except for CAT in female groups, where the assumption of multivariate normality was violated, and the nonparametric multivariate Kruskal–Wallis (MKW) test was used.

Values with *p* < 0.05 were considered significant.

## 3. Results

The clinical data as well as antioxidative parameters of OA and control subjects are presented in Table 1, Table 2 and Table 3.

There were no statistically significant differences in clinical parameters between Control and OA patient groups except for coefficient of variation (RDW-CV) values where an increase in OA subjects was observed. In contrast, antioxidant parameters were significantly decreased in OA group as compared to control. The only exception was CAT activity whose value in OA patients did not differ from that obtained for control (Table 1).

The comparison of clinical parameters values, performed with regard to gender, revealed that RBC, HGB, HCT and MCHC values were markedly decreased in females as compared to males, both in control subjects and OA ones. In the case of comparison of C-male vs. C-female, *p* < 0.001 was observed for the first three parameters and *p* = 0.00111 for MCHC. When OA-male group was compared with OA-female one, p values were 0.02170, 0.00018, 0.00280 and 0.01595 for RBC, HGB, HCT and MCHC, respectively. The obtained values are presented in Table 2.

The analysis of the obtained data concerning TAS values performed with regard to gender revealed a statistically significant depletion in OA-female group vs. the respective female control group (*p* = 0.01550), while in OA-male group no difference vs. male control was noted (*p* = 0.79759).

In the case of GPx, the analysis of the obtained results with regard to gender demonstrated that in both OA-female and OA-male groups GPx was decreased compared to respective control subgroups but these effects reached statistical significance only in the case of men (*p* = 0.00984), while in women the depression was insignificant (*p* = 0.56703).

In the case of SOD, the analysis of the obtained results with regard to gender revealed only an insignificant depletion, in both OA-female and male subgroups vs. the respective control subgroups (*p* = 0.58232 and 0.84746, respectively).

Unlike the comparison of the whole control with all OA patients, the analysis of the obtained CAT activity values with regard to gender proved that in OA females a significant depletion vs. the respective female control subgroup was noted (*p* = 0.03111), while in men a well-marked increase compared to male control was observed (*p* = 0.04073).

The results concerning the studied antioxidant parameters with regard to gender are collected in Table 3.

To exclude the effect of age on the observed differences in the studied parameters between OA patients and control group, the multi-way analysis of variance ANOVA/MANOVA or nonparametric multivariate Kruskal–Wallis (MKW) test was performed. It was shown that in males age had no influence on the differences between OA-male and C-male groups (TAS: F = 0.248, *p* = 0.624; SOD: F = 0.0005, *p* = 0.983; GPx: F = 0.017, *p* = 0.897; CAT: F = 0.941, *p* = 0.343). To unify the presentation of the obtained results, the same analysis was performed for females (OA-female and C-female), which resulted in the following values (TAS: F = 0.015, *p* = 0.985; SOD: F = 4.0396, *p* = 0.0235; GPx: F = 0.462, *p* = 0.633; CAT: F = 1.0681, *p* = 0.4380). The only significant result was the SOD increase along with age observed in females but, as in both males and females a decrease in SOD activity vs. the respective control was observed, this effect seems to be of little importance.

## 4. Discussion

The current study revealed impairment of antioxidant defense in OA patients, although in the case of the particular parameters the observed changes were disparate. Additionally, considerable differences between genders were depicted. These results are generally consistent with the majority of reports published by other scientists, although the opposite findings are also published [22,26,27]. For instance, Bhutia et al. found no significant correlation between knee OA severity and some chosen antioxidants (blood uric acid, SOD and GPx) [12].

Furthermore, similar to our study, in one of the available articles, the mean ages of the OA study group and control were significantly different (53.4 ± 2.3 vs. 41.0 ± 3.5; *p* = 0.001), but the authors found no correlations among the examined oxidant parameters and age [9].

It should be emphasized that, despite a rather extensive research on the oxidative processes in OA, the authors, even in the case of the recently published articles, performed no analysis considering gender, notwithstanding that numbers and compositions of the studied groups allowed it [7,8,30]. The only exception was the study published by Olszewska–Słonina et al. [25]. This seems rather odd, considering the differences in OA incidence between men and women.

The available data confirm the fact that oxidative changes caused by OA are a complex process, involving some parameters’ deterioration without the impairment of others.

Turkish scientists Sarban et al. reported that in OA subjects total antioxidative capacity in plasma was not different from the values obtained for healthy controls, whereas in erythrocytes GPx and CAT were found to be markedly decreased and SOD was not significantly changed [26]. According to Altindag et al., in subjects with knee OA from Turkey serum total antioxidant capacity and CAT were markedly depressed [31]. In OA subjects from India, erythrocyte CAT was depleted as compared with healthy controls. In contrast, erythrocyte GPx and SOD were enhanced [27].

Indian researchers Pinto et al. reported the results providing the next confirmation of the differences regarding the participation of particular antioxidants in the whole of oxidant processes occurring in OA course. The authors stated that plasma antioxidant activity was significantly decreased, erythrocyte SOD enhanced and erythrocyte CAT unchanged in subjects with knee OA as compared to healthy controls [22].

The differences among the particular elements of antioxidant defense both along the course of disease and during treatment of substances of antioxidant properties were also observed by Maghsoumi-Norouzabad et al. In a group of subjects with knee OA from Iran treated with acetaminophen + glucosamine, the additional 42-day administration of *Arctium lappa* L. (Burdock) root tea resulted in a significant increase in erythrocyte membrane-bound SOD as well as total antioxidants capacity in serum while erythrocyte membrane-bound GPx was only slightly enhanced. Interestingly, in control group without Burdock administration, after the study period no changes of total antioxidants capacity and SOD were noted, whereas GPx was markedly decreased [32].

The experiment performed by Olszewska-Słonina et al. revealed the existence of differences in antioxidant parameters in OA males and females from Poland. Red blood cell CAT and GPx were increased in the study group comprising both genders vs. control as well as in male and female subgroups vs. the respective male and female controls. SOD was unchanged in men and significantly decreased in women vs. male and female controls, respectively. Interestingly, when the analysis of the obtained results regarding SOD was made without considering gender, no difference vs. control was found. Additionally, the authors compared CAT, GPx and SOD activities in erythrocytes of OA patients prior and 10 days after surgery (arthroplasty). No significant differences in GPx and CAT were recorded, both in all study group as well as in male and female subgroups, while SOD was markedly increased in all three cases. However, this effect was more distinct in males [25]. The presented results confirm the necessity of applying the gender-dependent analysis in experiments involving OA subjects.

The lack of a direct relationship among particular antioxidants, observed in the current study, can also be confirmed by the outcomes reported by Barker et al. In a study performed on a group of 56 subjects with OA of the knee from the USA, divided into three subgroups considering serum vitamin D level (deficient, adequate and sufficient), they observed that, along with the vitamin D increase, serum α- and β-carotene were insignificantly but clearly enhanced while plasma ascorbic acid exhibited no distinct tendency and serum α-tocopherol remained unchanged [19]. On the other hand, Bhattacharya et al. revealed a significant enhancement of erythrocyte SOD, CAT and GPx concomitant with a well-marked lipid peroxidation (evaluated by an assay of its marker, malonyl dialdehyde) decrease in patients with OA of the knee from India supplemented with vitamin E (three months, 200 mg/day) [18].

The oxidative processes were also found to be involved into action of such pharmaceutical agents applied in OA cases as nonsteroidal anti-inflammatory drugs: celecoxib, meloxicam and ibuprofen. Turkish researchers Burak Cimen et al. reported that in OA patients undergoing 21-day treatment with those drugs, erythrocyte antioxidant potential and SOD activity were significantly decreased when compared to values observed before therapy, whereas CAT and GPx remained unchanged [33].

The involvement of the course of oxidative processes in OA development was also confirmed in animal model studies.

In experimental OA rabbits, an increase in nitric oxide (NO), concomitant with depression of SOD was observed in the serum. Additionally, these effects were alleviated by the glucosamine sulfate treatment, a pharmacological agent applied in OA patients [34].

According to Aborehab et al., in rats with experimental knee OA, a significant decrease in serum SOD was observed. Additionally, treatment with preparations of two herbs containing components of antioxidant properties, namely ginger and curcuma, alleviated this effect [5]. Similarly, in rats with OA developed as a consequence of experimental diabetes, serum SOD activity was markedly decreased [13]. Furthermore, Duan et al. revealed a significant depletion of SOD in serum of post-traumatic knee OA mice [14].

However, in another study performed on rats with experimental OA, an increase in plasma SOD concomitant with depletion of plasma CAT was noted [3].

In contrast, Adeyemi and Olayaki revealed a well-marked depletion of plasma SOD in rats with experimental knee OA, while plasma CAT remained unchanged [35].

The presented literature data as well as the results of the current study provide an undeniable evidence of a marked involvement of oxidative stress and changes of antioxidant defense in the development of OA. On the other hand, as far as the particular antioxidants are concerned, the lack of consensus among different authors’ outcomes may be observed. Such inconsistency could be, at least in part, caused by the fact that the studied subjects belonged to different races, came from different countries or continents and obviously presented different dietary habits. The relationships between diet and vitamin levels in organism in OA development have already been reported, although the results are not consistent [10]. All these factors could influence the obtained results, as genetic factors have been found to impact OA development. The differences in changes of enzymatic antioxidants could also be attributed to the development of any adaptive mechanisms under oxidative stress conditions. Maneesh et al. suggested that a significant SOD increase observed in OA patients, concomitant with GPx and CAT decrease might be regarded as such a mechanism [21]. A similar effect was observed in the present study in OA male subgroup—a well-marked enhancement of CAT could constitute a compensatory mechanism towards a significant GPx decrease as these two enzymes have a similar role as the antioxidants detoxifying hydrogen peroxide. In some part, this observation could be of some relationship with the less OA incidence observed in males. The possibility of the existence of any compensatory mechanisms in joint fluid was also studied by Regan et al. but the obtained results did not confirm such an assumption [20].

### Limitations

The study was performed on the group of OA patients and control subjects without osteoarthritis, other inflammatory processes or tumor. However, the numbers of OA patients and controls were different. Furthermore, there was a considerable difference in age between C-male and OA-male groups, but the multi-way analysis of variance ANOVA/MANOVA revealed no influence of age on differences in the studied parameters in men.

## 5. Conclusions

Obtained results suggest the possibility of existence of some compensatory mechanisms towards OA-related oxidative stress in men. Moreover, as the studies performed with regard to gender of OA subjects are scarce, further research seems to be needed; first, with the aim of clarifying the question of higher OA incidence in females; and second, to contribute to development of new therapy strategies in OA cases by the application of antioxidants as adjuvant agents. For instance, as some studies, including the present one, showed a decrease in a seleno-dependent enzyme GPx, the supplementation with selenium preparations could be considered. However, considering the lack of consistency of the available data, this issue requires more accurate and thorough study.

## Figures and Tables

**Table 1:** Clinical data and antioxidative parameters of OA (osteoarthritis) patients and control subjects.

Parameters	Control n = 21	OA Patients n = 67	*p*
Clinical Parameters			
WBC (10^9^/L)	6.57 ± 1.24	7.09 ± 1.80	0.21738 (t)
RBC (10^12^/L)	4.55 ± 0.64	4.47 ± 0.51	0.52731 (t)
HGB (g/dL)	14.0 (12.4–15.2)	14.0 (12.2–14.5)	0.22108 (U)
HCT (%)	39.7 ± 4.9	39.2 ± 4.1	0.60931 (t)
MCV (fL)	87.1 ± 3.2	88.0 ± 4.9	0.41259 (t)
MCH (pg)	29.7 ± 1.4	29.7 ± 2.0	0.97653 (t)
MCHC (g/dL)	34.2 ± 1.2	33.7 ± 1.1	0.10841 (t)
RDW-CV (%)	13.1 (12.6–13.7)	13.6 * (12.9–14.2)	0.03887 (U)
PLT (10^9^/L)	225 (195–254)	233 (204–261)	0.36004 (U)
PDW (%)	11.4 (10.8–13.9)	11.7 (11.0–13.0)	0.95706 (U)
MPV (fL)	10.0 (9.8–11.1)	10.3 (9.8–11.0)	0.70990 (U)
P-LCR (%)	28.9 ± 7.6	28.7 ± 6.8	0.91403 (t)
PCT (%)	0.25 (0.20–0.26)	0.25 (0.21–0.28)	0.51190 (U)
Antioxidant Parameters			
TAS (mmol/L)	4.70 ± 0.60	4.39 ± 0.53 *	0.02626 (t)
GPx (U/L)	675 ± 149	518 ± 176 ***	0.00042 (t)
SOD(U/mL)	127 ± 42	109 ± 32 *	0.04224 (t)
CAT (U/mL)	23.3(14.0–37.0)	22,1 (11.6–34.9)	0.82567 (U)

Values are mean ± SD for normally distributed variables while non-normally distributed ones are expressed as median and interquartile range. (t), *t*-Student test; (U), U Mann–Whitney test; * *p* < 0.05 vs. control; *** *p* < 0.001 vs. control. WBC, white blood cells; RBC, red blood cells; HGB, hemoglobin; HCT, hematocrit; MCV, mean corpuscular volume; MCH, mean corpuscular hemoglobin; MCHC, mean corpuscular hemoglobin concentration; RDW-CV, red blood cell distribution width, coefficient of variation; PLT, platelets; PDW, platelet distribution width; MPV, mean platelet volume; P-LCR, platelet large cell ratio; PCT, plateletcrit; TAS, total antioxidant status; GPx, glutathione peroxidase; SOD, superoxide dismutase; CAT, catalase.

**Table 2:** Clinical data of OA patients and control subjects with regard to gender.

Parameter	C-Male n = 11	C-Female n = 10	OA-Male n = 18	OA-Female n = 49
WBC (10^9^/L) (HSD)	6.30 ± 0.84 ^a^	6.86 ± 1.56 ^a^	7.32 ± 1.63 ^a^	7.01 ± 1.87 ^a^
RBC (10^12^/L) (HSD)	5.06 ± 0.23 ^a^	3.99 ± 0.45 ^b^	4.79 ± 0.47 ^a^	4.35 ± 0.48 ^b^
HGB (g/dL)	15.2	12.3	14.9	13.0
(KW)	(14.9–15.5) ^a^	(11.9–12.5) ^b^	(13.9–15.3) ^a^	(11.8–13.7) ^b^
HCT (%) (HSD)	43.4 ± 1.7 ^a^	35.8 ± 4.2 ^b^	42.3 ± 3.3 ^a^	38.1 ± 3.7 ^b^
MCV (fL) (HSD)	85.6 ± 1.7 ^a^	88.7 ± 3.6 ^a^	88.7 ± 4.7 ^a^	87.8 ± 5.0 ^a^
MCH (pg)	29.7	29.1	30.5	29.5
(KW)	(29.5–30.1) ^a^	(28.7–39.0) ^a^	(28.9–31.7) ^a^	(28.1–31.0) ^a^
MCHC (g/dL)	35.0	33.5	34.3	33.6
(KW)	(34.6–35.2) ^a^	(32.7–34.1) ^b^	(34.0–35.1) ^a^	(33.0–34.1) ^b^
RDW-CV (%)	12.6	13.7	13.5	13.7
(KW)	(12.4–13.1) ^a^	(13.1–14.2) ^a,b^	(12.9–14.2) ^a,b^	(12.9–14.1) ^b^
PLT (10^9^/L]	246	205	210	239
(KW)	(195–258] ^a^	(194–230] ^a^	(180–233) ^a^	(221–264) ^a^
PDW (%]	11.4	11.7	11.7	11.7
(KW)	(10.6–15.0) ^a^	(10.9–13.9) ^a^	(10.9–12.8) ^a^	(11.1–13.0) ^a^
MPV (fL]	10.0	10.0	10.1	10.3
(KW)	(9.5–11.4] ^a^	(9.9–11.1) ^a^	(9.8–10.9) ^a^	(9.8–11.0) ^a^
P-LCR (%]	25.5	26.4	26.2	28.0
(KW)	(21.6–35.8] ^a^	(24.3–34.8] ^a^	(23.4–31.7) ^a^	(24.9–33.2) ^a^
PCT (%]	0.25	0.24	0.21	0.26
(KW)	(0.18–0.26) ^a,b^	(0.20–0.27) ^a,b^	(0.19–0.24) ^a^	(0.23–0.28) ^b^

Values are mean ± SD for normally distributed variables while non-normally distributed ones are expressed as median and interquartile range. (HSD), Tukey’s honest significant difference test; (KW), Kruskal–Wallis test. The values in rows sharing the same superscript are not statistically significant. WBC, white blood cells; RBC, red blood cells; HGB, hemoglobin; HCT, hematocrit; MCV, mean corpuscular volume; MCH, mean corpuscular hemoglobin; MCHC, mean corpuscular hemoglobin concentration; RDW-CV, red blood cell distribution width, coefficient of variation; PLT, platelets; PDW, platelet distribution width; MPV, mean platelet volume; P-LCR, platelet large cell ratio; PCT, plateletcrit.

**Table 3:** Antioxidant parameters in OA patients and control subjects with regard to gender.

Parameter	C-Male n = 11	OA-Male n = 18	C-Female n = 10	OA-Female n = 49
TAS (mmol/L) (HSD)	4.41 ± 0.52 ^a^	4.61 ± 0.54 ^a,c^	5.02 ± 0.54 ^b,c^	4.31 ± 0.51 ^a^
GPx (U/L) (HSD)	715 ± 105 ^a^	482 ± 185 ^b,c^	630 ± 181 ^a,c^	532 ± 173 ^a,c^
SOD (U/mL) (HSD)	127 ± 39 ^a^	115 ± 22 ^a^	127 ± 48 ^a^	107 ± 35 ^a^
CAT (U/mL)	14.0	26.9	38.5	20.2
(KW)	(7.0–18.6) ^a^	(23.3–46.5) ^b,d^	(27.9–46.6) ^c,d^	(11.6–31.6) ^a,b^

Values are mean ± SD for normally distributed variables while non-normally distributed ones are expressed as median and interquartile range. (HSD), Tukey’s honest significant difference test; (KW), Kruskal–Wallis test. The values in rows sharing the same superscript are not statistically significant.
